# Supplementary material for: Dynalign II: common secondary structure prediction for RNA homologs with domain insertions
Source: Nucleic Acids Res. 2014 Nov 21;42(22):13939–48. doi: 10.1093/nar/gku1172 (PMC4267632; doi:10.1093/nar/gku1172)
Supplement: SUPPLEMENTARY DATA [file supp_gku1172_nar-02021-z-2014-File012.zip › manual/javadoc/comments/DrawingSource.html]

A package that encapsulates all the Java classes that handle structure and dot plot drawing.
  
  
There are no classes in this package.
Instead, all Java drawing framework classes are in the subpackages of this one.
This is because the drawing framework is backed by C++ classes, and these C++ classes are separated from the Java classes in the RNAstructure\_java\_drawing folder.
C++ classes cannot be documented with Javadocs, but are documented with Doxygen formatting.
  
  
The drawing classes can either be integrated into existing Java software or compiled to create self-contained drawing executables.
Either a structure drawing program (which can draw one structure file at a time) or a dot plot drawing program can be made.
The runners package is necessary for these self-contained programs, but not necessary when classes are integrated into existing software.
